# PERCEIVED SOCIAL ISOLATION AND HEALTH-RELATED QUALITY OF LIFE IN HEAD AND NECK CANCER

**DOI:** 10.1093/geroni/igz038.3552

**Published:** 2019-11-08

**Authors:** Janet H VanCleave, Jane M Fall-Dickson

**Affiliations:** 1 NYU Meyers College of Nursing, New York, New York, United States; 2 Georgetown University School of Nursing & Health, Washington, D.C., United States

## Abstract

Patients treated for head and neck cancer may experience impaired eating and talking that may affect their ability to undergo social activities. We conducted a secondary data analysis to explore: 1) prevalence of perceived social isolation, and 2) association between perceived social isolation and health-related quality of life (HRQoL) in patients with head and neck cancer. Data were collected during a clinical usefulness study of the Electronic Patient Visit Assessment (ePVA), a valid, reliable web-based patient-reported symptom measure for head and neck cancer. The study population consisted of 56 patients recruited during or after treatment for head and neck cancer. Perceived social isolation data were collected using the ePVA. HRQoL data were collected using the European Organization for Research and Treatment of Cancer (EORTC) QLQ-C30, a valid measure frequently used in the head and neck cancer population. Data analysis consisted of descriptive statistics and Student’s T-Test. The study population consisted primarily of persons > 60 years (mean age = 61.5 + 12), male (68%), White (77%), and receiving surgery, chemotherapy, radiation therapy or combination of these treatments (70%). Among participants, 36% reported that their current health situation negatively affected their social activities. Reasons for perceived social isolation included fatigue, feeling ill. Statistical analysis found that perceived social isolation was significantly associated with deceased HRQoL (t=5.3, p<.001). We conclude that participants in this sample treated for head and neck cancer were at risk for perceived social isolation, which has previously been reported to negatively influence cancer treatment outcomes.

